# A Modest Proposition to align Geriatrics and Long Term Care Medicine

**DOI:** 10.1186/1471-2318-8-29

**Published:** 2008-11-18

**Authors:** Vincent Mor, Paul R Katz

**Affiliations:** 1 Department of Community Health, Alpert Medical School, Brown University, USA; 2 University of Rochester School of Medicine and Dentistry, Canandaigua/Rochester, VA Medical Center, USA

Approximately 10 million Americans have long-term care needs,[1] including 2 million community-based elders who suffer impairments akin to those of the nation's 1.6 million nursing home residents.[2] In light of projections regarding the growth of the elderly population in the coming decades, particularly those over 85, the size of the elderly population requiring long term care who also have complex chronic conditions will likely double in 30 years.[3] While the population most in need of specialized geriatrics care is going to increase, the supply of geriatricians has been declining.

In contrast to the US, geriatricians in the United Kingdom (4 per 10,000 80+ year olds) work largely in the hospital environment and rarely enter nursing homes or manage home care services. In the US, in 2000 there were some 11 geriatricians per 10,000 80+ year old, including all geriatricians ever certified [4]. If we assume that there are about 10 million complex geriatric patients (about 25% of all older Medicare patients), and that they average 15 physician visits per year, were there as many as 10,000 US geriatricians, each with 1000 such patients, that would translate into 47 patient visits per working day!!! That is considerably more than the 20 minutes the average older patient spends with a physician these days. For geriatricians to be the primary care physicians for all complex, chronically ill older patients, the number of geriatricians would have to triple AND nurse practitioners or physicians' assistants would still have to be doing the majority of regular primary care to make this model viable.

Unfortunately, current trends do not bode well for geriatrics to be a specialty that inspires and attracts the best physicians willing to invest in the needs of the vulnerable population. The number of certified geriatricians is not increasing to meet demand, but declining each year and precious few young physicians are sitting for the boards and many of those initially "grand-fathered" into the specialty are not sitting for the re-examinations[5]. Compounding this problem is the fact that most health care professionals who will ultimately be caring for older adults during their careers have suboptimal training in geriatrics. Variability in the quality of care delivered to older adults in this country is a testament to these inadequacies.

In view of these "human resource" limitations we suggest that geriatrics in America focus on the smaller, more identifiable, long term care patient population by working individually with those patients and working corporately with the agencies providing those services. Nursing home medicine, terminal care and primary care to patients in congregate residential care settings all involve the kinds of skills in individual and populations/systems based medicine in which we should be training our geriatricians. Population medicine in this instance involves knowing about the systems of care, knowing that the problem identified in one patient is likely to be reflected in another in the same setting either because the problem reflects a systemic deficit in how patient care is provided or reflects the infectiousness of the bugs that are endemic to a given care environment.

We contend that the discipline of geriatric medicine must assume a leadership role, both privately and publically, in the long term care arena in order to improve the quality of care provided. Without medical leadership, assisted living will become a repeat of nursing home care and proprietary home health care will push procedures to maximize reimbursements in a shrinking pool of funds. Geriatricians must at once meet the challenge of providing high quality "individual" patient care to long term care recipients AND become conversant, even expert in, population based medicine, that views the entire resident population or home care case-load as the "patient". Addressing the patient specific needs while also facilitating care delivered by all providers working in the long term care organization is the real challenge but absolutely necessary.

Accomplishing such will require a restructuring of geriatrics into a specialty that is more organizationally aligned with long term care providers and committed to the individual patients they serve. While organizations such as the American Medical Directors Association (AMDA) have made great strides over the years in positioning long term care practice as a credible specialty, much more needs to be done. LTC practice models must be expanded and examined against accepted quality and cost benchmarks. One might imagine, for example, a properly organized LTC group practice to include the role of medical director of nursing homes, home health agencies and, to the extent that they exist, assisted living facilities. The bulk of the patients would be seen individually by the physicians in the practice and even short stay patients recovering from an acute episode but for whom a return to home is planned, could be seen by the practice in their role as the "SNFist" or, what some observers have termed the "Post acute care hospitalist". Hospital coverage of those patients in nursing home or assisted living and home care could be a rotating responsibility, which depending upon the volume, could be in a short stay hospital based, geriatric inpatient unit. This provides the opportunity to expose inpatient medicine residents in training to the principles of geriatrics at the individual as well as at the system level, with physicians modeling interaction and communication with discharge planning, rather than leaving that critical function to be someone else's job.

A focus on LTC does not mean that academic geriatrics should abandon its commitment to teach generalists the principles of geriatrics in a hospital setting as well as during nursing home, assisted living and home visit rotations. Indeed, inculcating other disciplines with a core geriatric knowledge base is at the heart of initiatives led by the American Geriatrics Society (AGS)[6]. However, organizational, reimbursement and the increasingly compartmentalized aspects of hospital care threaten the ongoing viability of this initiative. In the end, the resources of each of the specialties that serve adult patients must be brought to bear to bring about meaningful and sustained change that translates into optimal care of older adults at the hospital bedside.

If this new paradigm is to succeed, it is essential that we banish the image of the "nursing home hack"; the tired, compromised physician acquiescing responsibility for the medical care of frail elders in nursing homes to the staff nurses and to administrators interested in efficiency. Closed staff arrangements can't be peopled by such individuals, they must be peopled by physicians with all the skills that geriatrics was designed to inculcate[7]. This is clearly the model used in the "premier" facilities in the country and it is a model that could make an appreciable difference in the quality of care long term care patients receive.

We contend that one of the reasons that long term care has acquired such a terrible reputation is because of the absent physician; medicine has abdicated responsibility for patient advocacy in the long term care context. One couldn't have imagined a hospital in the 1970's without a chief of staff, medical director and organized medical staff with an influence on hospital practices and approaches. However, in spite of acuity that now rivals hospitals of that earlier era, that is how many nursing homes and home health agencies have operated, delegating the operation of the doctor's workshop to others so that the doctor doesn't really have to attend.

That is no longer a viable model; no longer acceptable. Physician leadership is essential if we are to ameliorate the myriad quality problems facing the vulnerable elderly in long term care. These are our patients; we owe them a functional definition of the role of geriatrics that is committed to them.

## Pre-publication history

The pre-publication history for this paper can be accessed here:


